# Increased Event-Related Potentials and Alpha-, Beta-, and Gamma-Activity Associated with Intentional Actions

**DOI:** 10.3389/fpsyg.2016.00007

**Published:** 2016-01-22

**Authors:** Susanne Karch, Fabian Loy, Daniela Krause, Sandra Schwarz, Jan Kiesewetter, Felix Segmiller, Agnieszka I. Chrobok, Daniel Keeser, Oliver Pogarell

**Affiliations:** ^1^Department of Psychiatry and Psychotherapy, Ludwig-Maximilians-UniversityMunich, Germany; ^2^Department of Child and Adolescent Psychiatry, Psychotherapy and Psychosomatic Medicine, Ludwig-Maximilians-UniversityMunich, Germany; ^3^Institute for Medical Education, Ludwig-Maximilians-UniversityMunich, Germany

**Keywords:** intentional action, event-related potentials, voluntary selection, EEG, oscillatory activity

## Abstract

**Objective:** Internally guided actions are defined as being purposeful, self-generated and offering choices between alternatives. Intentional actions are essential to reach individual goals. In previous empirical studies, internally guided actions were predominantly related to functional responses in frontal and parietal areas. The aim of the present study was to distinguish event-related potentials and oscillatory responses of intentional actions and externally guided actions. In addition, we compared neurobiological findings of the decision which action to perform with those referring to the decision whether or not to perform an action.

**Methods:** Twenty-eight subjects participated in adapted go/nogo paradigms, including a voluntary selection condition allowing participants to (1) freely decide whether to press the response button or (2) to decide whether they wanted to press the response button with the right index finger or the left index finger.

**Results:** The reaction times were increased when participants freely decided whether and how they wanted to respond compared to the go condition. Intentional processes were associated with a fronto-centrally located N2 and P3 potential. N2 and P3 amplitudes were increased during intentional actions compared to instructed responses (*go*). In addition, increased activity in the alpha-, beta- and gamma-frequency range was shown during voluntary behavior rather than during externally guided responses.

**Conclusion:** These results may indicate that an additional cognitive process is needed for intentional actions compared to instructed behavior. However, the neural responses were comparatively independent of the kind of decision that was made (1) decision which action to perform; (2) decision whether or not to perform an action).

**Significance:** The study demonstrates the importance of fronto-central alpha-, beta-, and gamma oscillations for voluntary behavior.

## Introduction

Executive functions can be seen as a set of cognitive abilities, e.g., planning, adaptive responses to changing environmental requirements, flexible responses, working memory, inhibition of responses, and selection between response alternatives. Executive functions refer to the many skills required to prepare for and execute complex behaviors (Ozonoff et al., 2004). Dysfunctions in the executive system impair the capability to analyze, plan, prioritize, schedule, initiate and complete an activity in a timely manner (Hosenbocus and Chahal, 2012). The psychopathology of many psychiatric diseases seems to be influenced by impairments of the executive system and are considerably associated with functional outcomes, disability and specific problem behaviors (Royall et al., 2002). Executive dysfunction has been linked to divers psychiatric conditions (Robinson et al., 2009), especially to attention deficit/hyperactivity disorder (ADHD) and to autism spectrum disorder (e.g., Happé et al., 2006; Hosenbocus and Chahal, 2012).

Fundamental aspects of executive functions are intentional actions. Intentional processes do not rely on obvious external stimuli but are self-generated, e.g., self-initiated movement and internally generated action plans. It is assumed that decisions are needed to produce intentional behaviors which are not stimulus driven (Brass and Haggard, 2008). By contrast, externally guided actions are influenced by sensory cues. Functional magnetic resonance imaging (fMRI) studies have demonstrated an association of voluntary selection processes and fronto-central areas (Turken and Swick, 1999; Ridderinkhof et al., 2004; Rushworth et al., 2007), including medial frontal areas, the supplementary motor area (SMA), the anterior cingulate cortex (ACC), and the dorsolateral prefrontal cortex (DLPFC) (Frith et al., 1991; Hyder et al., 1997; Jueptner et al., 1997; Lau et al., 2004b; Walton et al., 2004; Forstmann et al., 2006; Karch et al., 2009), the superior parietal lobule and the intraparietal sulcus (Forstmann et al., 2006; Karch et al., 2009).

Electrophysiological studies focusing on voluntary processes have demonstrated a fronto-centrally located negativity after about 200 ms (N2) and a positive deflection about 300 ms after the presentation of the task (P3; Karch et al., 2009, 2010a). In addition, the combination of electrophysiological and functional MRI results in a simultaneous EEG/fMRI study showed that the N2 amplitude was predominantly associated with BOLD responses in medial and lateral frontal brain areas, whereas functional variations of the P3 seemed to be related to both lateral frontal activities and parietal responses (Karch et al., 2009, 2010a). The function of the N2 is not yet clear: various studies focusing on executive functions demonstrated that the N2 is supposed to be a correlate of conflict detection (Van Veen and Carter, 2002), response inhibition (Falkenstein et al., 1999; Bruin et al., 2001; Bekker et al., 2004) or the detection of an endogenous mismatch process (Näätänen and Picton, 1986). The P3 seems to be associated predominantly with attention processes and the processing of information (Donchin and Coles, 1988; Kramer and Strayer, 1988; Polich and Kok, 1995), the selection between action alternatives (Gajewski et al., 2008) as well as response inhibition.

Analyses of intention-related variations in different frequency ranges are rare: one study revealed pronounced activity in high frequency ranges (>30 Hz; gamma band response) during intentional actions (Karch et al., 2012). Overall, numerous studies have demonstrated that cognitive processes, e.g., objects recognition, attention, and memory can modulate gamma band activity (Tiitinen et al., 1993; Yordanova et al., 1997; Debener et al., 2003; Herrmann and Demiralp, 2005). Increased gamma band activity can be found, for example during the concentration on auditory information as well as in subjects focusing attention on motor response preparation (Makeig, 1993; Yordanova et al., 1997) and selective attention (Tiitinen et al., 1993, 1997).

For the participation of higher association areas slower frequency ranges such as theta and alpha seem to play an important role (Klimesch, 1999; Basar et al., 2000). For example, memory processes seem to be related to *alpha activity* (8–12 Hz; Busch and Herrmann, 2003; Herrmann et al., 2004): responses in the alpha frequency range increase with increasing memory load (Schack and Klimesch, 2002; Busch and Herrmann, 2003). *Theta activity* (5–7 Hz) is also believed to be associated with hippocampal neurons and is often found during memory recall (Tesche and Karhu, 2000; Klimesch et al., 2001; Buzsaki, 2002). Altogether, the synchronous occurrence of theta/alpha/beta/gamma activity indicates the existence of distributed oscillatory systems which are interwoven with sensory and cognitive functions (Basar et al., 2000). Oscillations may act as communication networks through large populations of neurons (Basar et al., 2000).

In the current literature, decreased oscillations in cortical recordings are found in most psychiatric pathologies: a decrease of delta activity in almost all diseases, as well as frequency shifts in alpha- and the lower frequencies were recorded (Basar et al., 2015). However, there are paradoxical cases with increased oscillations, e.g., increased beta activity in patients with bipolar disorder, or an increase of gamma activity during cognitive loading in patients with schizophrenia (Basar et al., 2015). Overall, there is great evidence that gamma oscillations associated with cognitive processes are modulated in various psychiatric diseases, including ADHD (e.g.; Yordanova et al., 2001; Karch et al., 2012), schizophrenia (e.g., Leicht et al., 2010, 2015; Basar et al., 2015; Senkowski and Gallinat, 2015) as well as subjects at high risk for psychosis (Leicht et al., 2016), autism spectrum disorders (Stroganova et al., 2015), bipolar disorder (Ozerdem et al., 2010; Basar et al., 2015) and Alzheimer’s disease (Basar et al., 2015). It is assumed that impairments reflect disturbed information processing and an interruption of normal neuronal synchronization, e.g., caused by a dysfunctional GABA/glutamate system. It has been suggested that these processes contribute to impairments in the integration of cognitive and affective information (Ozerdem et al., 2010). Brass and Haggard (2008) proposed a model in order to distinguish different aspects of intentional action: the decision about which action to execute (*what* component), the decision about when to execute an action (*when* component), and the decision about whether to execute an action or not (*whether* component; Brass and Haggard, 2008). The *what* component can be addressed when participants can choose between various response alternatives (Botvinick et al., 2001; Nachev et al., 2007). The rostral cingulate zone and the pre-SMA seem to be especially related to the *what* component (Lau et al., 2004b; Walton et al., 2004; Krieghoff et al., 2009). The *when* component is related to the time-point of decision (Cunnington et al., 2003; Lau et al., 2004a). The superior medial frontal gyrus probably could be more clearly activated in the timing component (*when*; Krieghoff et al., 2009). In daily life subjects often have to decide on their own whether they should act or not. However, the *whether* component has hardly been investigated so far; a specific region in the fronto-median cortex might be related to these processes (Brass and Haggard, 2007).

The aim of the present study was to examine electrophysiological responses associated with intentional actions. Especially the influence of different aspects of voluntary actions (1) the decision about which action to execute (*what* component) (2) the decision to act or not (*whether* component) on electrophysiological responses will be addressed. We hypothesized that voluntary selection processes are related to enhanced N2 and P3 amplitudes in fronto-central brain areas (e.g., Näätänen and Picton, 1986; Gajewski et al., 2008). In addition, alpha-, beta, and gamma-band activity is supposed to be increased in frontal areas during intentional actions compared to externally guided responses.

## Materials and Methods

### Subjects

Twenty-eight healthy male subjects without any neurologic or psychiatric diagnosis (rated by a standardized questionnaire) participated in the EEG experiment. We included only males because several former studies demonstrated a gender effect for electrophysiological responses (Deldin et al., 1994). Several questionnaires were used to determine their actual mental state, e.g., the Beck Depression Inventory (BDI; Beck and Steer, 1987). One participant was excluded from any further analysis because the BDI score was higher than cut-off (cut-off > 14). Hence, 27 participants (aged between 20 and 34 years; mean age: 24.0 ± 2.71 years; mean BDI score: 2.70 ± 2.49) were included in the EEG analysis. Participants were recruited from an academic environment (education: mean: 16.4 ± 1.79 years; verbal intelligence: mean: 118.4 ± 8.55). The executive abilities of participants were not examined before the participation in the study.

The sample was randomly divided into two sub-samples; these groups were instructed to carry out two different versions of the same task (see *paradigm;* paradigm +/- and paradigm R/L). The two groups did not differ regarding age (paradigm +/-: number of participants: 14; mean age: 24.8 ± 3.31; paradigm R/L: number of participants: 13; mean age: 23.2 ± 1.64; *p* = 0.139), education (paradigm +/-: mean: 16.6 ± 1.96 years; paradigm R/L: mean: 16.3 ± 1.64 years; *p* = 0.631) and verbal intelligence (paradigm +/-: mean: 117.5 ± 9.21; paradigm R/L: mean: 119.3 ± 8.1; *p* = 0.593).

The study was approved by the local ethics committee of the Ludwig-Maximilians-University Munich. The investigation was carried out in accordance with the Declaration of Helsinki. Written informed consent was obtained from each participant after procedures had been fully explained. Each subject was paid €20 for participation in the study.

### Procedure, Paradigm, and Analysis of Behavioral Data

**FIGURE 1:**
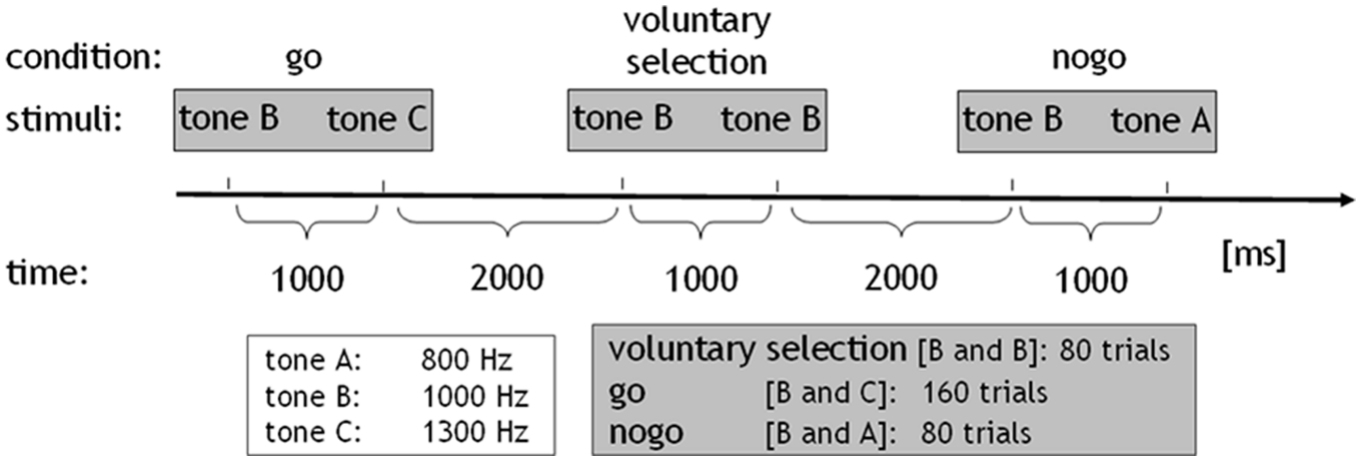
**Paradigm: sinusoidal tones of three differential pitches were presented (duration: 50 ms, pressure level: 100 dB).** The tones were presented in pairs at intervals of 1000 ms. The interval between trials lasted 2000 ms. The *go condition* comprised the combination of the middle-frequency tone (tone b: 1000 Hz; cue stimulus) followed by the high-frequency tone (tone c: 1300 Hz). In the *nogo task*, the cue stimulus was followed by a low-frequency tone (tone a: 800 Hz). In the *voluntary selection condition*, the cue stimulus was followed by the tone with a same frequency. The *go condition* was presented 160 times, *nogo*, and *voluntary selection* were presented 80 times.

All subjects performed an adapted go/nogo task where auditory stimuli consisted of sinusoidal tones (duration: 50 ms, pressure level: 100 dB) of three differential pitches, delivered binaurally via headphones. Tones were presented pairwise at intervals of 1000 ms. The interval between trials lasted 2000 ms. The *go condition* comprised the combination of a middle frequency tone (1000 Hz; cue stimulus) followed by a high frequency tone (1300 Hz). Subjects were instructed to press a response button with their right index finger and respond as quick as possible after the stimuli were presented, while minimizing any errors. In the *nogo task* the tone with the middle frequency (cue stimulus) was followed by a low frequency tone (800 Hz). During this condition, the prepared behavioral response was to be inhibited. In the *voluntary selection condition*, the cue stimulus was followed by the tone with an identical frequency (1000 Hz; *selection*; information about the paradigm see also Karch et al., 2009, 2012). Instructions regarding the voluntary selection condition differed between the two versions of the paradigm: in the first version, participants were instructed to freely decide whether to press the response button (*selection+*) or not (*selection-*) during the voluntary selection task (*paradigm +/-*). Participants were asked to decide separately in each trial of the *voluntary selection* condition whether they wanted to respond or not. Subjects were told that the ratio *selection+*/*selection-* should be approximately equally often. In addition, subjects were asked not to count how often they pressed the button and not to alternate between button press and not press. In *paradigm R/L*, two response buttons were provided. Subjects were instructed to decide whether they wanted to press a response button with the left (*selection_L)* or the right (*selection_R)* index finger. Participants were instructed to press the response button with the right and left index finger more or less equally often without counting the responses (see **Figure 1**).

In addition, both paradigms included a passive listening task which served as control condition. During the control condition, the tone with the low-frequency was presented first, indicating that no behavioral response was necessary regardless of which tone was presented next (*control condition*: 800–1000 Hz). All conditions were presented in pseudo-randomized order. The *go* condition was presented 160 times, the other conditions were presented 80 times, with an interstimulus interval of 3 s. Prior to the EEG session, all subjects received a practice block in order to ensure that the instructions had been fully understood (see also Karch et al., 2009, 2012).

Auditory stimuli were generated using the Presentation software package (version 14.2) and conducted via a set of headphones placed over the subjects’ ears. Participants kept their right index finger mounted on the button of the response box. During paradigm R/L, subjects were also instructed to keep their left index finger on the second response button.

### Behavioral Data

Reaction times, errors of omission (during *go task*) and errors of commission (during *nogo condition* and *passive listening task*) were recorded with the Presentation software. Any response delayed by more than 1500 ms after the stimulus was counted as error during the *go* condition. During the *voluntary selection* condition of paradigm +/-, behavioral responses during the interval 0–1500 ms after stimulus presentation were counted as *selection+*; trials without behavioral response within the first 1500 ms after stimulus presentation were counted as *selection*-. In paradigm R/L, responses with the right and the left index finger were recorded during the *voluntary selection* task. The mean reaction times were calculated separately for go and voluntary selection. Behavioral data (response times; error rates) were compared between conditions with ANOVA (within subject factors of paradigm +/-: *go*; *selection+*; within subject factors of paradigm R/L: *selection*; *go*). In addition, *t*-tests were calculated in order to examine differences regarding reaction time, percentage of correct responses and the error rate between paradigm +/- and paradigm R/L.

### EEG Acquisition and Data Analysis

The EEG was recorded with 32 electrodes (Neuroscan Synamps) using an electrode cap; Cz served as reference. Electrodes were positioned according to the International 10/20 system including the following electrodes: Fz, Cz, Pz, Fp1, Fp2, F3, F4, F7, F8, C3, C4, Cp5, Cp6, P3, P4, P9, P10, T5, T6, T3, T4, O1, O2, A1, A2, EOG, T1, T2, Fc5, Fc6, Fc1, Fc2. Data were collected with a sampling rate of 1000 Hz and without any filter during acquisition. Impedances were maintained below 5 kΩ. Participants were asked to stay calm and keep their eyes closed during the task. Recording took place in a sound-attenuated and electrically shielded room.

Pre-processing and data analyses were done with the Vision Analyzer Software (Brain Products, Munich). A common average reference was used. EEG data were filtered with a 1 Hz high-pass filter (slope 24 dB/oct), a 100 Hz low-pass filter (slope 24 dB/oct); a notch filter was not used. Eye-blinks were detected automatically and corrected using the correction of Graton & Coles using Fp2 as reference. EEG data were segmented into 2000 ms epochs time-locked to the onset of the second stimulus of each pair of tones, separately for each different condition (*voluntary selection, go, nogo*). The sampling epoch commenced 1000 ms before the presentation of the second tone that indicated which task was to be performed. An amplitude criterion (±70 μV) was used for artifact rejection involving *Fz*, *Cz*, and *Pz*. Baseline correction was done using the 200 ms interval before the second stimulus of each pair of tones. ERP wave-shapes were averaged separately for *go, nogo, voluntary selection condition*. Trials with incorrect responses were rejected prior to averaging. All wave-shapes included at least 30 averages.

In the paradigm +/- 94.2% of *go* trials (*M* = 150.7 trials), 94.8% of the *nogo* trials (*M* = 75.8) and 96.6% of the *voluntary condition* (*M* = 77.3 trials) were included on average for the analyses. Concerning the R/L paradigm 94.9% of *go* trials (*M* = 151.8 trials), 96.9% of the *nogo* trials (*M* = 77.5 trials) and 91.8% of the *voluntary condition* (*M* = 73.4 trials) were included on average for the analyses.

### Statistics

SPSS 18.0 program was used for statistical analysis. The significance level was 0.05, *p*-values between 0.05 and 0.1 were marked as a trend.

#### Event-Related Potentials

ERPs (N2 and P3) were examined at fronto-centro-parietal electrodes (Fz, Cz, Pz). The N2 was defined as the largest relative minimum of the ERP in the search window of 160–230 ms. The P3 was defined as the largest relative maximum of the ERP 230–550 ms after the presentation of the respective task. ANOVAs were run on the maximum ERP-amplitude in each search window (N2, P3) with two within subject factors *task* (*voluntary selection, nogo, go*) and *electrode position* (Fz, Cz, Pz). Because ANOVAs are not robust to violations of sphericity we checked for each within subject factor whether Mauchly’s test was significant. If so, the Greenhouse-Geisser corrected values for any effects involving this factor were reported. *Post hoc t*-tests were used in case of significant within subjects factors in order to analyze which task conditions and electrodes differed significantly from each other. Based on 3 × 3 task conditions, nine different tests were performed. The results of the *t*-tests were Bonferroni corrected.

#### Wavelet-Analysis

Evoked alpha-/beta- and gamma-activity were calculated using a complex Morlet wavelet transformation [see also (Herrmann et al., 1999; Mulert et al., 2007)]. The wavelet transformation was performed on averaged ERPs to reveal the phase-locked evoked fraction of the alpha-, beta-, and gamma-activity. As a first step, the frequency range from 1 to 60 Hz was divided into 40 frequency steps (distributed on a logarithmic scale) for each subject (Morlet parameter *c* = 5; continuous wavelet transformation; Morlet complex wavelet). In the next step, for each participant separate parameters were calculated for alpha (frequency range 8.06–12.09 Hz; mean: 10.08 Hz), beta (frequency range 20.16–30.25 Hz; mean: 25.21 Hz), and gamma frequencies (frequency range 32.27–48.40 Hz; mean: 40.34 Hz; see also Karch et al., 2012).

Alpha/beta/gamma power was identified at Fz, Cz, and Pz in the time-frame 0–500 ms after the presentation of the second tone of each pair of tones. The length of the interval was adapted to the waveform of the oscillatory responses. Amplitudes were detected automatically using the Brain Vision Analyzer-Software Version 1.05 (see also Karch et al., 2012). ANOVAs were employed to test for differences between electrode position and task condition, as well as between paradigms (paradigm +/- vs. paradigm R/L).

## Results

### Behavioral Results

The results are shown in **Table 1**. Mean response times were significantly longer in *voluntary selection* trials than in *go* trials in paradigm +/- [*F*(1,13) = 101.553; *p* < 0.001] and paradigm R/L [*F*(1,12) = 31.321; *p* < 0.001]. The percentage of responses was significantly increased in the *go* compared to the *voluntary selection* condition in paradigm +/- [*F*(1,13) = 200.96; *p* < 0.001] and in paradigm R/L [*F*(1,12) = 16.78; *p* = 0.001]. The error rate did not differ significantly between tasks [*F*(1,12) = 6.783; *p* = 0.523].

**Table 1:** Behavioral data: the response times were significantly longer in *voluntary selection* trials than in *go* trials in paradigm +/- and paradigm R/L.

	Paradigm +/-		Paradigm R/L	
	*M*	*SD*	*M*	*SD*
**Reaction time (ms)**				
Go	516.0	116.93	568.7	127.49
Voluntary selection	811.0	178.95	850.2	242.42
**Percentage of responses (%)**				
Go	97.95	1.81	97.31	3.34
Voluntary selection	56.34	10.63	94.38	3.18
**Error rate (%)**				
Responses during nogo or control	3.74	2.41	2.23	2.50

*In addition, the percentage of responses was significantly increased in the go compared to the voluntary selection condition in both versions. The error rate did not differ significantly between tasks. M, Mean value; SD, standard deviation; ms, milliseconds; %, percentage.*

The comparison of behavioral data of paradigm +/- with those of paradigm R/L revealed comparable reaction times (*p* = 0.273) and comparable percentages of correct responses (*p* = 0.543) during *go*. In addition, the reaction time did not differ significantly during the *voluntary selection* task (*p* = 0.635). The percentage of responses was significantly higher in paradigm R/L compared to paradigm +/- (*p* < 0.001). Participants used the left button in 53.7% of voluntary selection trials (reaction time: *M* = 861.6 ms) and the right button in 45.4% of trials (reaction time: *M* = 840.3 ms). Error rates were comparable in both groups (*p* = 0.122).

### Comparison of ERPs During *go, Nogo, and Voluntary Selection* Condition

**FIGURE 2:**
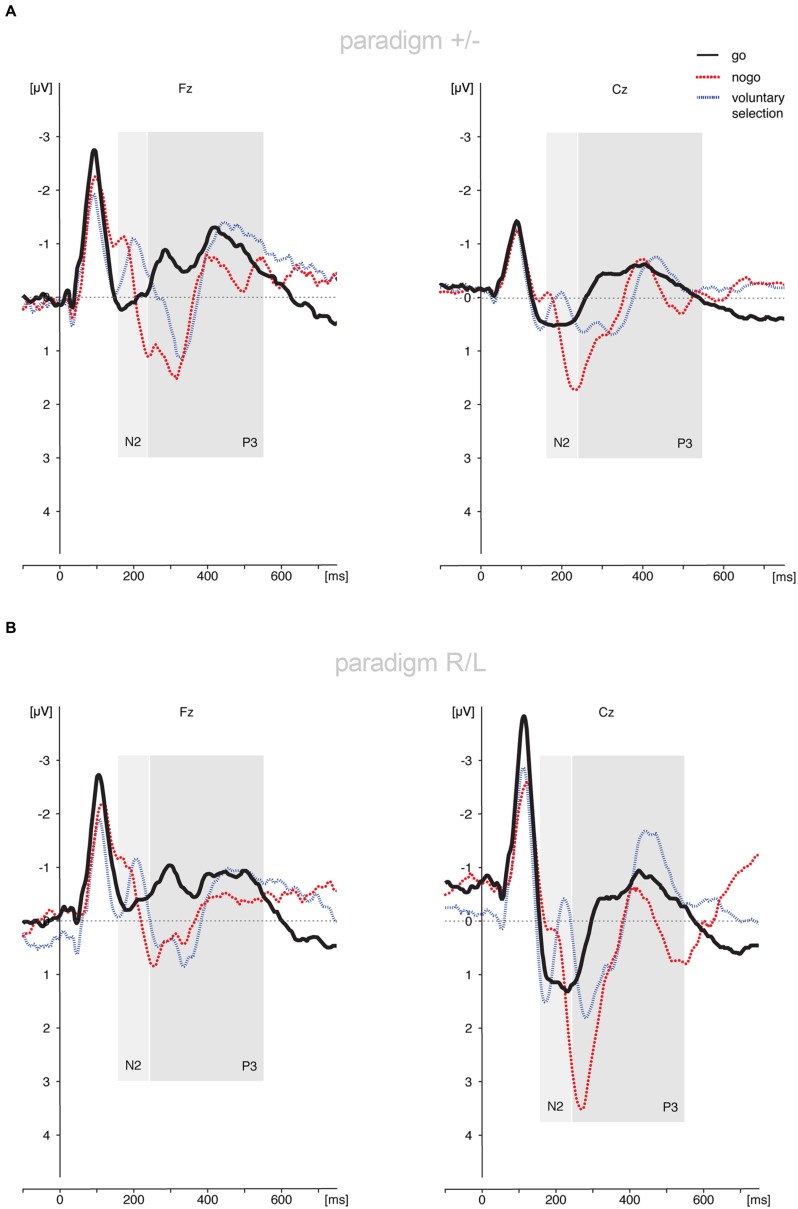
**Auditory evoked potentials of healthy controls. (A)** ERPs concerning the paradigm +/-; **(B)** ERP results regarding the paradigm R/L. Subjects showed increased fronto-centrally located N2 amplitudes during the *voluntary selection task* and *nogo condition* compared to *go.* The P3 amplitude was located in fronto-central brain areas during the *nogo* condition and the *voluntary selection* condition. Abbreviations μV, microvolt; ms, milliseconds.

**FIGURE 3:**
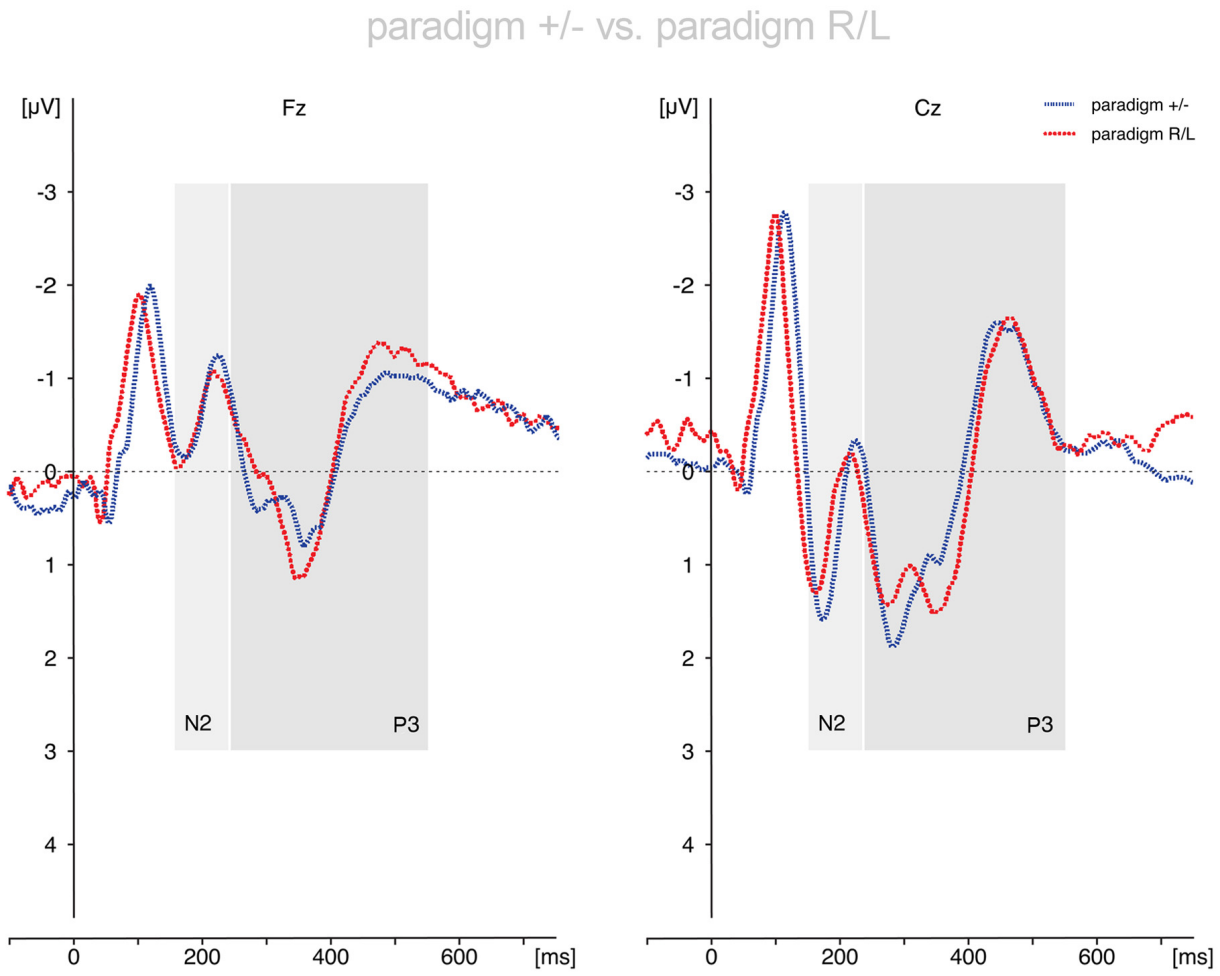
**Comparison of the *voluntary selection*-related responses during paradigm +/- and paradigm R/L.** The ERP results differed only marginally between paradigms. Abbreviations μV, microvolt; ms, milliseconds.

Results are shown in **Figures 2** and **3**.

#### Paradigm +/-

##### N2

Regarding the N2-amplitude, in paradigm +/- the main effect of *condition* (*go*, *nogo*, *voluntary selection*) [*F*(2,26) = 6.376; *p* = 0.006] and the main effect of *electrode position* (Fz, Cz, Pz) [*F*(2,26) = 4.922; *p* = 0.034] turned out to be significant. The interaction between *electrode position* and experimental *condition* was not significant [*F*(4,52) = 1.918; *p* = 0.162]. Further analyses revealed that the N2 was significantly less pronounced in the *go* task compared to the *voluntary selection* condition (*p* = 0.015) as well as the *nogo* (*p* = 0.032). The N2 did not differ between *voluntary selection* and *nogo* (*p* = 1.0). When focusing on the localisation of the N2 (*electrode position*), the N2 was enhanced in Fz compared to Cz (*p* = 0.010). The comparison of Fz and Pz (*p* = 0.280) as well as Cz and Pz (*p* = 1.0) did not reveal significant differences.

##### P3

The results of the P3-amplitudes showed significant main effects of *condition* [*F*(2,26) = 23.267; *p* < 0.001] and *electrode position* [*F*(2,26) = 3.437; *p* = 0.047] as well as a significant interaction effect (*condition* × *electrode position*) [*F*(4,52) = 9.392; *p* < 0.001]. The P3 amplitude was increased in *nogo* trials compared to *go* trials (*p* < 0.001) and the *voluntary selection* condition (*p* = 0.008). Apart from this, *voluntary selection* associated P3 amplitudes were increased compared to those of the *go* task (*p* = 0.005). Regarding the position of the electrodes, there was a significantly increased P3 in Cz compared to Fz (*p* = 0.020). The P3 in Fz and Pz (*p* = 1.0) as well as Cz and Pz (*p* = 0.171) did not differ significantly.

*Post hoc* tests of the interaction effect revealed significant differences in Fz and Cz between *go* and *nogo* (Fz: *p* = 0.001; Cz: *p* < 0.001), *go* and *voluntary selection* (Fz: *p* = 0.021; Cz: *p* = 0.015) as well as *nogo* and *voluntary selection* (Fz: *p* = 0.035; Cz: *p* = 0.004). The differences in Pz were not significant (*go* vs. *nogo*: *p* = 0.385; *go* vs. *voluntary selection: p* = 0.205; *nogo* vs. *voluntary selection: p* = 1.000).

#### Paradigm R/L

##### N2

The assessment of the N2 amplitude demonstrated that the main effects of *condition* [*F*(2,24) = 7.163; *p* = 0.004] and *electrode position* [*F*(2, 24) = 12.230; *p* < 0.001] were statistically significant; the N2 amplitudes were more negatively in Fz compared to Cz (*p* < 0.001) and Pz (*p* = 0.008). The N2 did not differ between Cz and Pz (*p* = 1.0). In addition, the N2 was less pronounced in *go* trials compared to *nogo* trials (*p* = 0.041) and *voluntary selection* trials (*p* = 0.041). The results of *nogo* and *voluntary selection* were comparable (*p* = 1.0). The interaction effect (*condition* × *electrode position*) was not significant [*F*(4,48) = 0.866; *p* = 0.491].

##### P3

The P3-amplitudes differed significantly between *conditions* [*F*(2,24) = 11.218; *p* < 0.001] and *electrode positions* [*F*(2,24) = 8.574; *p* = 0.007]. Apart from that, the interaction effect (*condition* × *electrode position*) was significant [*F*(4,48) = 6.001; *p* = 0.004]. *Post hoc* tests showed smaller P3 amplitudes in *go* compared to *nogo* (*p* = 0.008) and *voluntary selection* (*p* = 0.008); the P3 in nogo and voluntary selection was comparable (*p* = 0.152). Regarding the localisation, the P3 was decreased in Fz compared to Cz (*p* = 0.005) and Pz (*p* = 0.003); the difference between Cz and Pz was not significant (*p* = 0.602).

*Post hoc* tests of the interaction effect revealed significant differences in Fz and Cz between *go* and *nogo* (Fz: *p* < 0.001; Cz: *p* = 0.023), *go* and *voluntary selection* (Fz: *p* = 0.006; Cz: *p* = 0.023). The difference between *nogo* and *voluntary selection* was not significant for Fz (*p* = 0.368) but Cz (*p* = 0.045). The differences in Pz were not significant (*go* vs. *nogo*: *p* = 1.000; *go* vs. *voluntary selection: p* = 0.608; *nogo* vs. *voluntary selection: p* = 1.000).

#### Comparison of the Results of Paradigm +/- and Paradigm R/L

##### N2

The N2-amplitudes differed significantly between *conditions* [*F*(2,50) = 12.947; *p* < 0.001] and *electrode positions* [*F*(2,50) = 12.997; *p* < 0.001]. The interaction effects *condition* × *electrode position* reached trend level [*F*(4,100) = 2.533; *p* = 0.076]. The interactions *condition* × *group* [*F*(2,50) = 0.181; *p* = 0.835], *electrode position* × *group* [*F*(2,50) = 0.106; *p* = 0.828] and *condition* × *electrode position* × *group* [*F*(4,100) = 0.190; *p* = 0.868] were not statistically significant. The *group* effect (paradigm +/-; paradigm R/L) was not statistically significant (*p* = 0.921). *Post hoc* analysis revealed enhanced N2 amplitudes during *nogo* trials compared to *go* trials (*p* = 0.001) and in *voluntary selection* trials compared to *go* trials (*p* = 0.001), but no differences between *nogo* and *voluntary selection* (*p* = 1.0). With respect to the localisation of the N2 amplitudes, the results showed an increased N2 amplitude in Fz compared to Cz (*p* < 0.001) and Pz (*p* = 0.007) whereas the N2 in Cz and Pz did not differ significantly (*p* = 1.0).

##### P3

Regarding the P3-amplitude there were significant main effects of *condition* [*F*(2,50) = 32.524; *p* < 0.001] and *electrode position* [*F*(2,50) = 10.831; *p* < 0.001]. In addition, the interaction *condition* × *electrode position* [*F*(4,100) = 14.495; *p* < 0.001] was significant. By contrast, the interactions between *condition* × *group* [*F*(2,50) = 0.382; *p* = 0.632], *electrode position* × *group* [*F*(2,50) = 2.239; *p* = 0.117] and *condition* × *electrode position* × *group* [*F*(4,100) = 0.292; *p* = 0.883] were not significant. In addition, the *groups* did not differ significantly [*F*(1,25) = 0.010; *p* = 0.921].

*Post hoc t*-tests indicated a significantly increased P3 amplitude during *nogo* trials compared to *go* trials (*p* < 0.001) and *voluntary selection* trials (*p* = 0.001). In addition, *selection*-related P3 amplitudes were increased compared to *go*-associated responses (*p* < 0.001). The P3 was increased in central areas compared to frontal regions (Cz > Fz; *p* < 0.001); the difference between Fz and Pz (*p* = 0.150) as well as Cz and Pz (*p* = 0.082) was not significant.

*Post hoc* tests of the interaction effect revealed significant differences in Fz and Cz between *go* and *nogo* (Fz: *p* < 0.001; Cz: *p* < 0.001), *go* and *voluntary selection* (Fz: *p* < 0.001; Cz: *p* = 0.002) as well as *nogo* and *voluntary selection* (Fz: *p* = 0.009; Cz: *p* < 0.001). The differences in Pz were not significant (*go* vs. *nogo*: *p* = 0.237; *go* vs. *voluntary selection: p* = 0.077; *nogo* vs. *voluntary selection: p* = 1.000).

### Results of the Wavelet-Analysis

**FIGURE 4:**
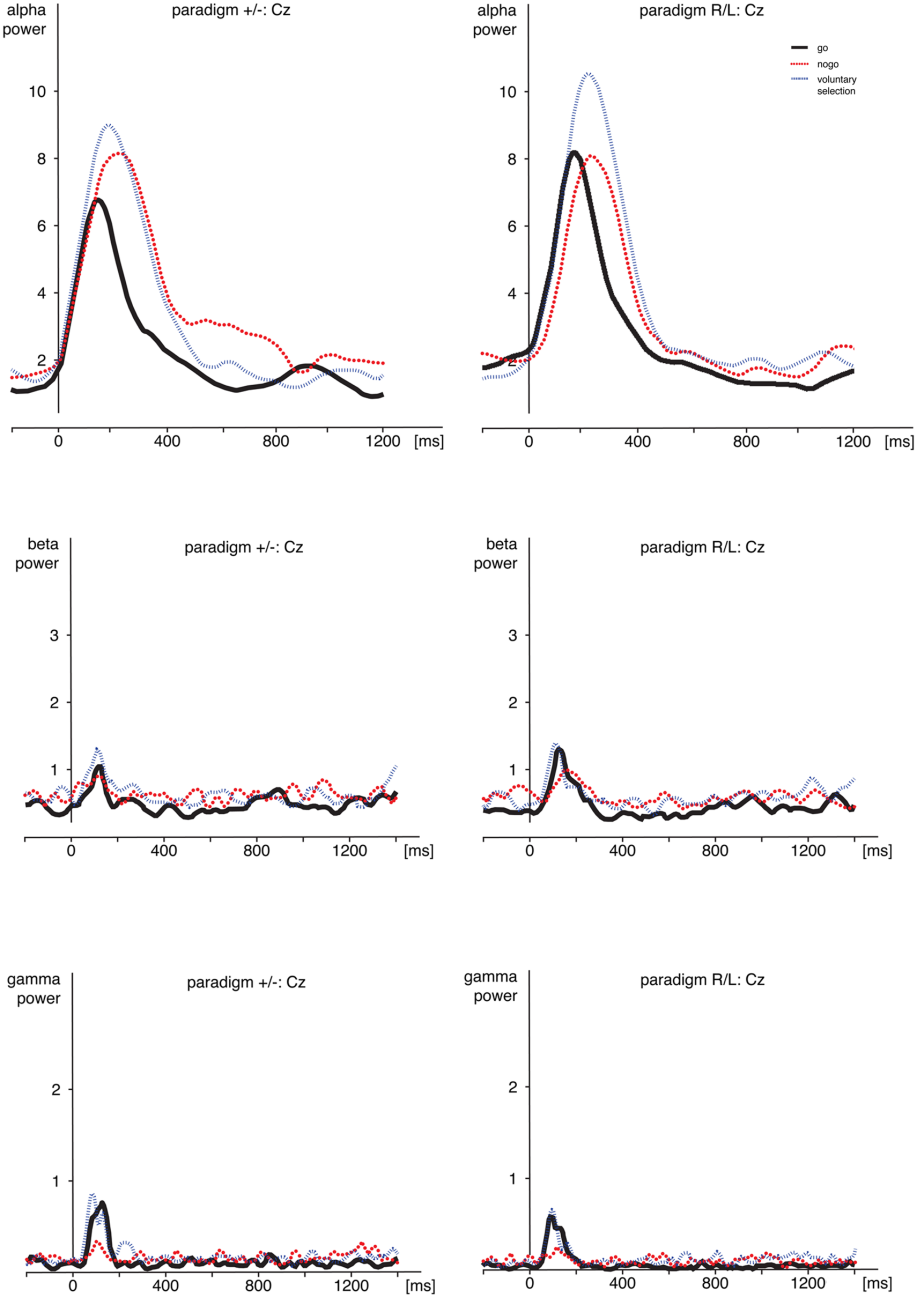
**Time frequency analyses.** Comparison of alpha-, beta-, and gamma-power during *go*, *nogo*, and *voluntary selection* in Cz. Activity was increased during *voluntary selection* compared to *go* and *nogo* condition. ms, milliseconds.

The results of the wavelet-analysis are shown in **Figure 4** and **Table 2**.

**Table 2:** Wavelet analysis: mean value of alpha, beta, and gamma power during *go*, *nogo*, and *voluntary selection* in paradigm +/- and paradigm R/L.

	Paradigm +/-			Paradigm R/L		
	Fz	Cz	Pz	Fz	Cz	Pz
**Alpha power**						
Go	5.48	7.05	4.06	5.36	8.35	5.30
Nogo	7.51	9.48	6.27	6.15	9.08	5.61
Voluntary selection	5.80	9.64	6.53	6.68	10.75	6.54
**Beta power**						
Go	1.26	1.28	0.96	1.28	1.61	1.17
Nogo	1.26	1.37	1.23	1.42	1.50	1.15
Voluntary selection	1.39	1.57	1.29	1.49	1.65	1.41
**Gamma power**						
Go	1.06	1.23	0.31	0.74	0.91	0.35
Nogo	0.88	0.62	0.49	0.72	0.53	0.50
Voluntary selection	1.63	1.39	0.63	1.33	1.04	0.55

#### Alpha Frequency Range

Regarding alpha activity, responses related to paradigm R/L did not differ significantly from those related to paradigm +/- [*F*(1,25) = 0.120; *p* = 0.732]. The main effect of *condition* [*F*(2,50) = 6.538; *p* = 0.003] was significant and demonstrated increased responses during *voluntary selection* compared to *go* (*p* = 0.005) but not between voluntary selection and nogo (*p* = 1.0) or nogo and go (*p* = 0.078).

The alpha response was significantly increased in the central area compared to frontal and parietal areas (Cz compared to Fz (*p* < 0.001), and Cz compared to Pz (*p* < 0.001) [*F*(2,50) = 25.477; *p* < 0.001]; Fz compared to Pz did not differ significantly (*p* = 1.0). The interactions were not significant {condition × group: [*F*(2,50) = 1.546; *p* = 0.223]; electrode position × group [*F*(2,50) = 0.365; *p* = 0.696]; electrode position × condition: [*F*(4,100) = 1.938; *p* < 0.110]; condition × electrode position × group: [*F*(4,100) = 0.703; *p* = 0.592]}.

*Post hoc* tests indicated increased alpha power during voluntary selection compared to go (*p* = 0.005); the results of voluntary selection and nogo (*p* = 1.0) and nogo and go (*p* = 0.078) did not differ.

#### Beta-Frequency Range

We did not find any difference in beta power between paradigm +/- and paradigm R/L [*F*(1,25) = 1.401; *p* = 0.248]. The beta activity differed significantly between *conditions* [*F*(2,50) = 8.952; *p* < 0.001] with increased responses during the *voluntary selection* condition compared to the *go* condition (*p* < 0.001) and compared to the *nogo* task (*p* = 0.031), but no differences between *go* and *nogo* (*p* = 0.857). In addition, there was a significant main effect of *electrode position* [*F*(2,50) = 6.666; *p* = 0.003] with an increased activity in Cz compared to Pz (*p* = 0.002) whereas the results of Fz and Pz (*p* = 0.231) and Fz and Cz were comparable (*p* = 0.282).

All interactions were not significant [*condition* × *group*: *F*(2,50) = 0.688; *p* = 0.507; *electrode position* × *group*: *F*(2,50) = 0.229; *p* = 0.796; *condition* × *electrode position: F*(4,100) = 0.403; *p* = 0.806; *condition* × *electrode position* × *group: F*(4,100) = 0.917; *p* = 0.457].

#### Gamma Frequency Range

Gamma power related to the paradigm +/- and the paradigm R/L did not differ significantly [*F*(1,25) = 0.741; *p* = 0.398]. However, the main effects of *condition* [*F*(2,50) = 10.492; *p* < 0.001] and *electrode position* [*F*(2,50) = 11.378; *p* < 0.001] as well as the interaction between *condition* and *electrode position* [*F*(4,100) = 5.232; *p* = 0.001] were significant.

Gamma activity was more pronounced in *voluntary selection* trials compared to *go* trials (*p* = 0.001) and *nogo* trials (*p* = 0.002); gamma activity of *go* and *nogo* did not differ (*p* = 0.654). Gamma activity was especially located in frontal and fronto-central areas: differences between Fz and Pz (*p* < 0.001) as well as Cz and Pz (*p* = 0.013) associated gamma activity were significant. Differences between Fz and Pz were not significant (*p* = 1.0). The interaction effects *condition* × *group* [*F*(2,50) = 0.331; *p* = 0.675], *electrode position* × *group* [*F*(2,50) = 0.591; *p* = 0.557] and *condition* × *electrode position* × *group* [*F*(6,306) = 0.158; *p* = 0.959] were not significant.

## Discussion

Intentional actions are supposed to be purposive and goal-directed as well as endogenously controlled (Brass and Haggard, 2008). In addition, attention is required in intentional actions and they offer a choice between alternatives (Jahanshahi, 1998). The aim of the present study was to distinguish electrophysiological correlates (ERPs; alpha, beta, gamma power) of intentional actions and externally guided actions. In addition, we compared neurobiological findings of different aspects of intentional actions: (1) “*what*” component comprising the decision, which action to perform; (2) “*whether*” component, which refers to the decision, whether or not to perform an action (Brass and Haggard, 2007, 2008). For that purpose, subjects were instructed to decide voluntarily whether they wanted to respond by button press or not (paradigm +/-; “*whether*” component). Another experimental task included the decision to respond with their right or their left index finger (paradigm R/L; “*what*” component).

With regard to the behavioral data, we found increased reaction times associated with intentional actions (*voluntary selection*) compared to externally guided responses (*go*). These findings are in line with the results of earlier studies using the same paradigm (Karch et al., 2009, 2010b). In addition, the responses of the participants during *voluntary selection* were carefully monitored by the instructor in order to prevent that the responses were systematically (e.g., alternating response and no response). Taking this information together may indicate that an additional cognitive process is essential for the processing of intentional responses compared to stimulus-driven actions. Reaction times did not differ, irrespective of the kind of intentional process (decision what to do or whether to respond or not).

In the present study, intentional processes were associated with a fronto-centrally located N2 and P3 potential. The N2 and P3 amplitude were increased during voluntary selection processes compared to instructed responses (*go*). The findings of some former studies regarding voluntary selection are similar to those of the present study (Karch et al., 2009, 2010a,b). By contrast, the results of Waszak et al. (2005) regarding electrophysiological correlates of intentional and stimulus-based actions differ considerably from the findings of the present study. Their results demonstrated that electrophysiological responses occurred much earlier in the intention-based condition compared to the stimulus-based condition. The response-locked lateralised readiness potential remained relatively invariant between conditions. The authors assumed that the results provide evidence for two different modes of action selection: one mode seems to be stimulus-driven, the other seems to be mainly intention-driven (Waszak et al., 2005). The differences between the results of the present study and the study of Waszak et al. (2005) could be influenced by differences regarding the experimental paradigm.

Information about the functional meaning of the N2 and P3 is inconsistent: there is some evidence that the N2 is associated with the selection of a response and influences subsequent stages of processing reflected in the P3. Unexpected revisions of the response program seemed to delay and enhance the N2 (Gajewski et al., 2008). Forstmann et al. (2007) suggested that the choice-related N2-P2 complex might reflect early sensory-perceptual processing, whereas the P3 is associated with the evaluation of the stimulus (Forstmann et al., 2007). The authors assumed that a choice between several task sets invokes medial frontal activity. Apart from that, a number of other brain regions seemed to be relevant for choices including parieto-occipital areas (Forstmann et al., 2007).

The ability to localize generators of ERP components is limited because of the low spatial resolution of EEG recordings. The results of a simultaneous EEG and functional MRI study using the voluntary selection paradigm showed that N2-related neuronal responses were mainly associated with medial and lateral parts of the frontal cortex. By contrast, the P3 was predominantly related to enhanced neuronal responses in lateral frontal brain areas and the temporo-parietal junction (Karch et al., 2010a). This may indicate that the frontal cortex is involved at an earlier stage than temporal and -parietal regions (Karch et al., 2010a).

Medial frontal areas, including the rostral cingulate zone, have already shown to be involved in the control of voluntary behavior as well as conflict monitoring, error detection and decision making (Jenkins et al., 2000; Botvinick et al., 2001; Garavan et al., 2002; Lau et al., 2004b, 2006; Nachev et al., 2005; Forstmann et al., 2006; Karch et al., 2009). These concepts might be partly overlapping: Krieghoff et al. (2011), for example, suggested that response conflict and volition represent two sides of the same coin and that there is no will without “conflicting” ideas (Krieghoff et al., 2011).

N2 and P3 amplitudes were not only detected during intentional actions but also during response inhibition. The N2 amplitude during *nogo* and *voluntary selection* did not differ significantly. By contrast, fronto-central P3 amplitudes were increased during response inhibition compared to both voluntary behavior and stimulus-dependent responses. Our findings of pronounced nogo N3 and P3 potentials are in line with former studies: response inhibition processes were frequently associated with a fronto-centrally located N2 potential and P3 potential (Pfefferbaum et al., 1985; Kopp et al., 1996; Bruin et al., 2001; Donkers and van Boxtel, 2004; Smith et al., 2004; Bekker et al., 2005). The nogo N2 appeared to be located in medial frontal regions (Bekker et al., 2005). It is assumed that the N2 is relevant for the suppression of incorrect response tendencies (Falkenstein et al., 1999) and could be associated with the rare presentation of stimuli (Donkers and van Boxtel, 2004; Bartholow et al., 2005), or is linked to stimulus classification (Ritter et al., 1983) as well as conflict (Randall and Smith, 2011). The frontally located P3 seemed to be more clearly associated with response inhibition and could be an indicator of both cognitive and motor inhibition (Smith et al., 2008), alternatively it reflects the cancelation of a planned response (Randall and Smith, 2011).

Concerning oscillatory responses, we detected increased alpha, beta, and gamma activity during intentional actions compared to instructed responses (*go*). Apart from that, beta- and gamma-power were more pronounced during voluntary responses compared to the inhibition of behavioral responses. Increased oscillatory activity was predominantly located in frontal and fronto-central areas. These results may indicate that oscillatory responses might be more helpful to further distinguish functional correlates of voluntary responses and response inhibition.

In general, there is a broad consensus that different kinds of oscillation denote different brain activity states and that oscillatory fluctuations across time are representative of the dynamic interplay between different cell types in various cortical and subcortical circuits (Buzsaki, 2006). The application of sensory or cognitive stimuli influences these responses. Oscillatory phenomena are strongly interwoven with sensory and cognitive functions: oscillatory processes could play a major role in relation to memory and integrative functions (Basar et al., 2000).

Especially gamma-band synchronization has attracted considerable interest over recent years because mechanistic roles have been proposed in phase coding, perceptual integration, and flexible routing of information in the visual system (Fries, 2009; Vinck et al., 2013), and furthermore because of its appearance in multiple cortical and subcortical structures (Fries, 2009). Former studies have demonstrated that oscillations in higher frequency ranges, especially gamma activity, are influenced by a various cognitive processes including object recognition, attention and working memory as well as the preparation of motor responses (Tiitinen et al., 1993; Yordanova et al., 1997; Engel et al., 2001; Debener et al., 2003; Basar-Eroglu et al., 2007). In addition, gamma activity increases with increasing task difficulty (Senkowski and Herrmann, 2002; Posada et al., 2003; Mulert et al., 2007). Midline areas, especially the dorsal part of the ACC and the medial frontal cortex, are assumed to be related to gamma-band responses (Mulert et al., 2007). These processes are assumed to be influenced by inhibitory interneurons and pyramidal cells (Bartos et al., 2007; Cardin et al., 2009; Sohal et al., 2009). Bosman et al. (2014) assumed that gamma band activity originates from the interplay between inhibition and excitation. Overall, gamma band oscillations support multiple cognitive processes rather than a single one. At a higher functional level, gamma-band oscillations seem to be influenced by visual attention, decision-making, response timing, motivation and short- and long-term memory (Bosman et al., 2014). They coordinate neuronal activity in hippocampal and neocortical networks (Kann et al., 2014). Cortico-cortical communication and the large scale integration of disturbed sets of neurons are needed for a well functioning cognitive ability and require synchronous neural gamma oscillations (Rodriguez et al., 1999).

EEG oscillations in the alpha and theta band reflect cognitive and memory performance (Klimesch, 1999). In addition, alpha band responses have been associated with working memory (Schack and Klimesch, 2002; Busch and Herrmann, 2003; Klimesch et al., 2005). Alpha activity was shown to grow with increasing memory load (Klimesch et al., 2005). Thalamo-cortical circuits as well as hippocampal areas are supposed to be relevant for the generation of alpha responses (Basar, 1998, 1999).

In addition, a recent review provides evidence that theta band activities over the mid-frontal cortex seem to reflect a common computation used for realizing the need for cognitive control (Cavanagh and Frank, 2014). Theta band processes may be used to communicate this need and subsequently implement such control across disparate brain regions. Thus, frontal theta is a compelling candidate mechanism by which emergent processes, such as ‘cognitive control,’ may be biophysically realized (Cavanagh and Frank, 2014).

The results of the present study are in line with the assumptions concerning the function of alpha, beta, and gamma oscillations: intentional responses might require pronounced cognitive process including cognitive control mechanisms as well as higher cognitive processes including decision-making in order to be effective. Overall, the results provide evidence that intentional actions might be more complex and seem to be related to cognitive control compared to instructed responses as well as response inhibition.

Intention-related variations in the alpha, beta, and/or gamma band activity seem to be measurable with the paradigm. This may provide the possibility to determine functional variations that are related with intentional actions in various neuropsychiatric disorders, e.g., in patients with ADHD or schizophrenia. It has been suggested that impairments regarding brain oscillations reflect disturbed information processing and a disruption in normal neuronal synchronization, e.g., caused by dysfunctional GABA/glutamate system, may contribute to deficits in cognitive and affective integration (Ozerdem et al., 2010). Basar et al. (2015) assume that oscillatory activity obtained by various input modalities are capable of displaying the relationship between any given neuropsychiatric disturbance and different neurotransmitter systems. In addition, brain oscillations may also show plasticity or compensation (Basar et al., 2015): a decrease in one frequency range may occur in parallel with the increase in a different frequency range.

In future, differences regarding intention-related neuronal responses between different neuropsychiatric disorders as well as the effect of psychotherapeutic interventions and pharmacological treatment on intention-related neuronal processes may be determined.

Another important aspect of the present study was a dissociation of neural correlates of different aspects of intentional actions. We did not find reliable differences regarding both ERPs and oscillatory responses: alpha-, beta-, and gamma-activity did not differ significantly between “whether” and “what” decisions. In addition, the N2 and P3 amplitudes were comparable. These results are somewhat surprising. However, up to now only a few studies exist that focus on this topic. Krieghoff et al. (2009) showed that the rostral cingulate zone is involved in the decision of which action to perform. By contrast, a part of the superior frontal gyrus in the paramedian frontal cortex seemed to be involved in the decision of “when” to perform action (Krieghoff et al., 2009). Brass and Haggard (2007) examined neural aspects of the “whether” component: in their study subjects were instructed to cancel an intended response at the last possible moment. Functional MRI results demonstrated the involvement of the dorsal fronto-median cortex (Brass and Haggard, 2007). To our knowledge, “what” and “whether” decisions have not been compared directly so far. In addition, these studies focused on functional differences concerning the localisation of the functional correlates of decisions. Differences regarding the localisation were not examined in the present study because of the low spatial resolution of electrophysiological responses. By contrast, differences regarding the functional meaning of the processes involved were addressed.

Altogether, the results of the present study indicate that intentional actions are related to fronto-centrally located N2 and P3 potentials. These responses seemed to be more pronounced than those related to instructed responses and the instructed inhibition of responses. In addition, alpha-, beta-, and gamma-band responses were increased during the voluntary selection between response alternatives, compared to instructed responses. These results suggest that an additional cognitive process is needed for intentional actions compared to instructed behavior. The neural responses were comparatively independent of the kind of decision that was made.

## Conflict of Interest Statement

The authors declare that the research was conducted in the absence of any commercial or financial relationships that could be construed as a potential conflict of interest.
